# Decoding exchange rate in emerging economy: Financial and energy dynamics

**DOI:** 10.1016/j.heliyon.2025.e41995

**Published:** 2025-01-16

**Authors:** Saif Ullah, Haitham Nobanee

**Affiliations:** a College of Management and Applied Sciences at Ziauddin University, Karachi, Pakistan; b College of Business, Abu Dhabi University, Abu Dhabi, 59911, United Arab Emirates; c Oxford Centre for Islamic Studies, University of Oxford, Oxford, OX3 0EE, UK; d Faculty of Humanities & Social Sciences, University of Liverpool, Liverpool, L69 3BX, UK

**Keywords:** Exchange rate, Foreign investments, Energy fuel prices, Stock market return. currency depreciation, Currency appreciation

## Abstract

This research paper examines the financial and energy-related factors influencing the exchange rate in Pakistan, focusing on currency depreciation or appreciation between July 2006 and June 2021. The Autoregressive Distributed Lag (ARDL) Bounds Test is employed to analyze both short- and long-run dynamics. The findings reveal that foreign direct investment (FDI) supports strengthening the exchange rate, addressing concerns about short- and long-term profitability. Energy factors, such as fuel prices, positively impact the exchange rate in both the short and long term. Conversely, stock market declines and rising inflation are associated with significant changes in the exchange rate, including currency depreciation. Interestingly, remittances exhibit a complex relationship with the exchange rate, potentially resulting in adverse effects if not managed effectively. The study provides balanced policy implications for sustainable financial and energy development in emerging economies.

## 1 Introduction

The exchange rate serves as a barometer for an economy, reflecting the relative strength or weakness of the currency in emerging markets [1]. Exchange rate change as currency depreciation is defined as the loss of value of a country's currency against foreign currencies. Currency depreciation is a critical issue in many countries worldwide. Therefore, modeling the exchange rate is crucial, whereby it is necessary to address the determinants of fluctuations in the rate with empirical evidence. The behavior of the exchange rate has enormous importance to an economy. Thus, it is essential to recognize the trends and problems of currency depreciation or appreciation as the exchange rate changes behavior, especially in a country like Pakistan that is facing such issues. Change in the exchange rate as currency depreciation or appreciation occurs due to lack of foreign direct investments, remittances, fuel prices for energy and production level enhancement, instability in stock market returns, and inflation.

Unfortunately, Pakistan is one of the countries that have suffered financial and energy instability and trouble maintaining stability, socio-economic conditions, and its international image. The fast change in the exchange rate as currency depreciation increases the cost of running newly established businesses, established companies, and ongoing energy production projects, which is a leading cause of reduced international market competitiveness of enterprises, growth, financial stability, and the overall development of the economy and the country. In Pakistan, importing many raw items like fertilizers, oil, food stuff, machinery, and other materials has severe consequences for exchange rates as currency depreciation. On the other hand [2], has suggested that a country's central bank or managing body of the exchange rate takes monetary steps to promote the management of the exchange rate system to attract FDI because the exchange rate factor influences the world's FDI and energy production projects. Furthermore, the connection between Foreign Direct Investment (FDI) and currency rates is complex, impacting long-term economic paths and structural changes, rather than just influencing short-term fluctuations [3]. The influence of foreign direct investment (FDI) on exchange rates is contingent upon the existing economic circumstances and settings, emphasizing the significance of context-specific studies in evaluating its effects on currency markets and overall financial stability [4].

In recent years, many countries have adopted an exchange rate system that is more flexible and open to the financial markets; therefore, the relationship between change in the exchange rate and stock return is noteworthy to explore [5]. Hence, the complex connection between stock market performance and exchange rates necessitates thorough analysis in the financial industry, particularly as globalization becomes more pronounced [6,7].

The link between energy price fluctuations and exchange rates is substantial, as the fluctuation of energy prices has a considerable and multifaceted effect on exchange rates in global markets. Nevertheless, there is still a lack of investigation in financial research about this particular field, and an in-depth understanding of the fundamental mechanisms and consequences is still required [8]. The relationship between energy prices and exchange rates encompasses the advantages and difficulties of incorporating energy investment objectives into financial and economic systems. This simultaneous impact highlights the importance of meticulous administration and strategic foresight to guarantee that environmentally friendly investments contribute to sustainability and economic stability.

Furthermore, the impact of remittances on exchange rates depends on increased demand for local currency, boost to foreign exchange reserves, economic stimulus, and market conditions. Remittances can contribute to currency appreciation by increasing demand for the local currency and foreign reserves. In addition, the relationship between inflation and currency rates is influenced by an intricate combination of factors and mechanisms. Examining the impact of inflation on exchange rate fluctuations is crucial since it reveals substantial implications for many economic participants, such as policymakers, firms, and investors. The research on FDI, market return, and energy with inflation can provide stakeholders with a comprehensive understanding of the broader economic effects and enhance the decision-making process by incorporating insights [7,9,10].

Past studies have mainly focused on the impact of currency depreciation on the GDP, political clashes, or the general effect of terrorism on the country. Some studies on developing countries, such as [11,12] have examined the war on terror's effect on the economy and money-related markets by concentrating on recognizing the role played by foreign and local investors. Additionally [13], explored the risk exposure of insecurity versus immaturity by focusing on the relationship between political violence risk terrorism and firm performance by examining firm-specific variables in Pakistan.

Foreign investments are financial activities that generate financial returns along with economic inclusiveness. These investments emphasize sustainability, resource efficiency, and renewable energy [14,15]. Foreign investments promote renewable energy investments, which impede the accomplishment of the sustainable development targets for 2030 [16]. The influence of foreign investments was also positive on economic performance [17], as foreign investments can increase demand for the local currency, strengthening the currency exchange rate. Foreign investments contribute and help stabilize the exchange rate by funding sustainable projects. Foreign investments foster sustainable development, promoting exchange rate stability and a stable currency. Foreign investments influence exchange rate trends by impacting financial market behaviors and altering investor preferences toward sustainable initiatives [18,19]. This highlights the crucial significance of finance in advancing sustainability and influencing economic indicators such as currency rates in global financial markets. However, they ignored the factors of foreign investment, financial markets, and energy prices in relation to the exchange rate. Hence, there is a need to explore the significance of determining the less-identified financial investment-markets-energy determinants of the exchange rate in Pakistan.

In this context, this study aims to determine the various elements that cause changes in exchange rates, such as currency depreciation or appreciation. The primary research aims are to examine and contribute to the nexus of financial investment-markets-energy related factors with the exchange rate in Pakistan from July 2006 to June 2021. The important factors that affect exchange rates, such as currency depreciation or appreciation, include foreign direct investment, stock market return, energy (fuel) prices, remittances, and inflation to provide sustainable and balanced growth policy recommendations by contributing to this domain.

This study provides significant contributing insights into the factors that influence exchange rates in emerging markets, particularly in the context of Pakistan. It adds valuable knowledge to the existing literature on this subject. Firstly, it expands the empirical framework by including less explored conventional financial factors, such as foreign direct investment (FDI), offering a comprehensive perspective on the economic forces affecting currency valuation. There is an effort to investigate the complex relationships between stock market returns and exchange rates, which are essential since they provide valuable insights into their significant effects on the global financial environment. Furthermore, the study provides detailed and subtle observations about the ever-changing connection between the performance of the stock market and fluctuations in exchange rates, an area that has not been extensively studied in the context of Pakistan. The incorporation of energy prices, specifically fuel, introduces an additional aspect of how reliance on energy affects the economic stability of a developing nation. Through analyzing these connections, the study not only addresses a notable deficiency in the field of regional financial research but also assists policymakers and economic strategists in developing more knowledgeable and efficient economic policies to handle fluctuations in exchange rates and promote long-lasting growth [20,21]. Moreover, remittances and inflation play a significant controlling role in the financial energy and exchange rate nexus. Hence, this endeavor can significantly influence scholarly discussions and pragmatic economic strategizing in developing economies. Comprehending such information is crucial for investors, politicians, and financial analysts as it helps them navigate the intricate connections that influence the financial ecosystem. Exploring the relationship among critical financial indicators can lead to a better understanding of the factors that influence how they interact with each other [6,7].

The relevant literature is reviewed in the next section, followed by the methodology section, in which the estimation techniques, data collection method, and justifications based on previous studies are discussed. Then, the results and discussion are presented. The last section concludes the study and offers suggestions for future research.

## 2 Literature review and hypotheses development

### 2.1 Dependent variables: exchange rate as currency depreciation and appreciation

The currency exchange value loss or increase against other nations' currencies, such as the U.S. $, is called currency depreciation or appreciation [11,22]. It increases the exchange rate due to supply and demand for money [23,24]. claims that a real exchange rate increase is real currency depreciation. Therefore, the bilateral real exchange rate (RER) has been introduced to rectify bias due to multilateral resistance. In Nigeria, [25]; in Vietnam, [19]; and in Bangladesh [26], have investigated the exchange rate and economy and reported consensus that to tackle the currency depreciation problem, the monetary and fiscal policies must be consistent [27]. expressed that exchange rate policy and capital flow management have stated that the exchange rate's stability is essential for a country's monetary policy goal.

The competitive situation in the international market, stocks, inflation, and import taxes are due to currency depreciation. These may be favorable in the short run, but ultimately, they lead to economic losses in the country [28,29]. [26] expressed concern over the relationship between the exchange rate and economic growth by employing a cointegration technique on an analytical framework to check the output response of the exchange rate as currency depreciation in Bangladesh. They found that exchange rate currency depreciation affects the production output, GDP economic development, policy, and public needs in the short and long run. Thus, the exchange rate behavior remarkably affects the economy and socioeconomic development [30].

In order to manage the exchange rate, a country's state bank or central bank holds foreign currencies, which refers to the foreign exchange reserve, and the reason for holding reserves is currency values [31]. Presently, domestic currency exchange rate depreciation lowers the expected return rate in foreign currency such as USD $ deposits. The foreign currencies deposited into the accounts of local banks from exporters were transferred to the Central Bank and changed into the local currency for usage. Mostly, foreign trade and investment are conducted in USD $ because of its status as the world's most accepted mode of currency exchange. When banks notice a decline in cash reserves, they have less money to lend for investment due to a drop in the savings ratio [32]. This situation impacts investment in the country experiencing capital flight of exchange rate depreciation or appreciation. To maintain their currency exchange value, countries use their foreign exchange reserves. For example, China uses a fixed rate, which pegs the value of its currency (yuan) to the US$. On the other hand, Japan and India use a floating exchange rate system [33].

Historically, the Pakistani Rupee was linked to the pound. Nonetheless, in 1971, it was dissociated from £ (pound) and was pegged to US$. Then, in 1982, the Government of Pakistan adopted a floating exchange rate mechanism. Furthermore, Pakistan introduced new financial reforms due to sanctions in the financial sector. These reforms somehow benefited Pakistan's economy and decreased the chink in the official and market rates. Besides, Pakistan adopted three multiple exchange rate systems because of atomic explosions. In the first system, the official rate was pegged to the US$ at a flat rate of the Rupee. Next is the Floating Interbank Rate (FIBR), also known as the quoted rate of commercial banks. Lastly, there is the composite exchange rate system, a combination of FIBR and the official rate system. Nonetheless, the use of these multiple exchange rate systems caused the creation of different bands. Moreover, the Pakistani Rupee was again pegged to the US$ in 1999 and 2000. Regardless, the Pakistani government removed the bands. Since then, the floating exchange rate mechanism has been used. The exchange rate in Pakistan showed an upward trend from 1982 until 2001 and then began to decline from the end of 2001–2005. After 2005, the rate continuously changed, and the country had a high exchange rate and became a stable country, but the currency was still unstable [11].

**Fig. 1:**
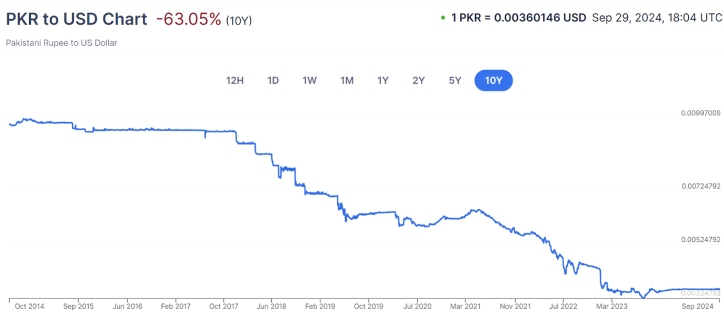
Trend Summary of the exchange rate of U.S. dollars per 1 Pakistani Rupee.

The exchange rate with currency depreciation issue has had a severe effect in recent times on the economy of Pakistan because the country's past policy had chosen to keep the Pakistani rupee overvalued, even though it had an adverse impact on the domestic economy. The Pakistani Rupee depreciated significantly, which caused several problems and hindered the country's economic growth. Furthermore, the Pakistani currency has drastically devalued against the US$ for many reasons, such as poor performance of the stock market, decrease in the foreign development investment (FDI), export reduction, terrorism, violent activities, weak governance, inflation etc. [26] stated that a growing balance of payments deficit and price rise had caused anxiety due to the country's macroeconomic conditions such as inflation and public scrutiny of the exchange rate [34,35]. have suggested that consistency in exchange rates kept currencies significantly appreciating. The summary of the trend of the exchange rate of U.S. dollars per 1 Pakistani Rupee is demonstrated in Fig. 1.

(Source: xe.com)

### 2.2 Independent variables financial and energy factors effect on exchange rate

In this study, exchange rate determinants include financial determinants, including FDI and stock market returns; energy determinants include fuel prices. Control factors included remittances and inflation. Each determinant is discussed in the following subsections based on theory, culminating in hypothesis development.

#### 2.2.1 Foreign direct investment (FDI) and exchange rate

FDI involves the transfer of capital from one country to another to establish a long-term interest in a business and development. FDI influences exchange rates (currency depreciation or application) due to a country's economic fundamentals, market conditions, and policy responses. FDI inflows can contribute to exchange rate stability under certain circumstances. FDI can also positively affect economic growth, trade, and investor confidence. Therefore, FDI, real exchange rate, imports, and remittances have been considered crucial from a growth perspective [36]. A long-run association was established between China's outward foreign direct investments, inflation, and exchange rate [3] from 1990 to 2017.

Commercial banks' investment in different currency deposits dominates the foreign exchange market. Therefore, FDI greatly contributes to the stability of the exchange rate [37]. In Pakistan [38], found a positive connection between FDI and sending countries' real output from 1974 to 2011; however, due to Pakistan's economic situation, FDI has turned out to be pro-cyclical. In a study by Ref. [8], the nexus of FDI and change in the exchange rate in Somalia between 1970 and 2010 was examined. This study revealed a negative and significant link between the exchange rate and FDI. Besides that [39], has investigated the relationship between the FDI and exchange rate instability and its effect on the one-belt one-road countries' international trade. They concluded that the FDI and exchange rate variability nexus could harmfully affect each other and international trade in developing countries [40,41]. have stated that developing economies rely on foreign capital and construct policies to attract FDI. Therefore, FDI and investment are critically linked to countries' exchange rates.

[42] have argued that the economies of emerging markets summon progressively more noteworthy weight to make foreign investors more vulnerable in foreign investments to the dangers connected with politically or monetary delicate governments, such as inflation, because political clashes are regular, especially in vast parts of Africa, Asia, and the Middle East. The exchange rate will also increase if overseas investment declines due to a lower money supply [43]. investigated the relationship between foreign investments and exchange rates and emphasized that the exchange rate is higher because of the negative impact of FDI and vice versa. Foreign investments' influence on currency exchange rates is growing due to the interplay of international financial movements. As countries progressively incorporate sustainability objectives into their economic policies, the rise in foreign investments has become a crucial determinant of currency valuations. FDI investments need to be converted at the current exchange rate, which affects the currency's strength [[44], [45], [46]]. One of the primary sources of money, which aids in economic growth and savings, is foreign investment. Foreign investments increase the supply of foreign currency, which helps reduce the exchange rate [37,47].

Moreover, the relationship between FDI and exchange rates is intricate, as evidenced by case studies conducted in countries such as China, Nigeria, Pakistan, and the United States, which shed light on this interconnection. For example, when the local currency strengthens relative to the USD, foreign direct investment (FDI) tends to decline [48,49], negatively affecting financial growth in countries such as Nigeria [50]. Furthermore, statistics from the United States show that FDI outflows are influenced by both the exchange rate effects between two countries and the exchange rate effects involving a third country. Typically, FDI inflows reduce when the currency of the country where the investment is being depreciated [51]. These varied results emphasize policymakers' need to consider these complex processes when formulating policies for sustainable economic growth. The relationship between foreign investments and exchange rates is multifaceted, and this dual impact underscores the necessity for careful management and strategic planning to ensure that the adverse effect does not erode the positive contribution of other sources to both the sustainability and economic stability of exchange rates.

H1 FDI has a significant positive effect on the exchange rate.

#### 2.2.2 Stock market return and exchange rate

A stock market is where trade and businesses are conducted through either a stock exchange or a counter market for the occupation of securities and company shares. It is also known as an equity market due to the sale and purchase of equities of listed companies [42,52]. [53] expressed concern over the association between the exchange rate and the equity market from 1983 to 1994 and found a bidirectional relationship in Japan. For Hong Kong, findings showed that the bidirectional determinant smoothed over the changes in the exchange rate to changes in stock prices; nevertheless, no causality was recorded for Singapore. On the other hand, based on the Ghana Stock Exchange (GSE) [54], showed a significant association between market return and exchange rate. Next, there was a negative relationship between stock return and a change in the exchange rate in Pakistan in the short run [55]. Lastly, in a study based on Pakistan and India [56], showed no association between the stock market return and exchange rate. Therefore, it is vital to study in-depth because of mixed findings and to determine the current updates.

The existing body of literature offers empirical evidence supporting the presence of a mutually beneficial and complex connection between stock returns and foreign exchange valuations such as [[57], [58], [59], [60], [61], [62]] have found that the performance of the stock market is influenced by changes in foreign exchange rates, financial ratio analyses, and the profitability metrics of companies. These studies emphasize a reciprocal relationship where stock market gains both influence and are influenced by changes in exchange rates, especially in the context of international capital movements. The relationship is further examined within macroeconomic indicators by considering the inflation rate. Empirical data suggests a notable negative effect on stock returns, especially in the short term [62]. Moreover, academic research has uncovered a reciprocal cause-and-effect relationship between financial performance indicators, such as Return on Assets and Debt to Equity Ratio, and stock market returns [58]. The insights from the relationship between stock performance and exchange rate changes are incredibly beneficial for portfolio managers involved in foreign investment initiatives. Understanding this information allows for a more detailed and comprehensive assessment of investment returns. It analyzes equities return measurements and exchange rate patterns to strengthen risk management techniques. This analysis emphasizes the need for advanced diversification and careful use of hedging measures to reduce the risks associated with currency exchange rate exposure to global investments.

H2 Stock market returns negatively influence the exchange rate.

#### 2.2.3 Energy (fuel prices) and exchange rate

Commodities like crude oil, gas, and fuel are traded in US$ in the global market, and an increase in the USD$ reduces the purchasing power of other currencies, which leads to the increase in the price of fuel, coal, oil, and LNG in terms of the local currency [63]. Hence, the influence of the exchange rate on the prices of commodities is complex [5]. [64] showed that fuel, oil, and gas prices significantly affected currencies' relative value. Besides that [65], has expressed concern over the relationship of fuel/oil as energy prices with the exchange rate and results revealed that in small open economies, fuel/oil prices are expected to impact the relative value of currencies exchange rate significantly. He suggested that changes in oil energy prices have substantial implications for the demand and supply of foreign exchange rates. Similarly [66], has demonstrated that changes in petrol prices significantly affect the exchange rates of other factors concerning the economy and inflation [67]. stated that exchange rate volatility significantly influences Nigeria's crude oil exportation.

A country's energy dependency, trade balance, inflation dynamics, and policy responses can affect the association between energy (fuel prices) and exchange rate. A comparison was made by Ref. [45] to measure the effects of gas and oil price hikes on the exchange rates to reveal that these exchange rate fluctuations are due to gas price shocks. The findings indicate that energy price hikes are associated with the appreciation of the Canadian dollar against the Euro and Japanese Yen. The prices of energy fuels, including essential commodities such as crude oil, natural gas, and electricity, significantly influence the exchange rates of many countries, as demonstrated by Ref. [62]. Empirical research highlights the significant correlation between energy price changes and currency valuation volatility [68]. Another recent investigation has shown that fluctuations in the prices of essential energy resources, such as crude oil, coal, and natural gas, have a consistent and lasting impact on exchange rates. This relationship is evident in short-term and long-term perspectives [69]. Furthermore, the relationship between global crude oil prices and the exchange rates of economies reliant on oil is complex and influenced by several factors and analytical approaches. Therefore, it is necessary to address energy source prices and exchange rate nexus, especially in emerging economies.

Financial assets, such as exchange-traded funds (ETFs) that specifically target energy sectors, significantly influence exchange rates. This is because they attract investments from institutional investors towards environmentally friendly activities, as evidenced by studies conducted by Ref. [70]. The relationship between the effectiveness of green policies and the impact of cross-border financial flows on currency prices is highlighted by this study [71]. Moreover, the volatility that arises from the green energy markets can affect other types of assets, impacting the dynamics of exchange rates [72]. Energy investments can enhance a country's currency by signaling a strong and sustainable economy. This view has the potential to increase the trust of foreign investors, which in turn can lead to an increase in the value of the national currency. This situation may arise if the investments are inadequately managed or if they reallocate resources from other crucial sectors of the economy, resulting in imbalances that worry investors. Nevertheless, energy-related green investments can decrease exchange rates, especially when they are perceived as economically hazardous [5,73].

H3 Fuel price decreases have a positive impact on exchange rates while an increase in Fuel prices vice versa.

### 2.3 Control variables: remittance and inflation effect on exchange rate

#### 2.3.1 Remittance and exchange rate

Pakistan is one of the largest labor-exporting countries in the South Asia region [74]. Remittance in this country grew by 5.5 % in 2018 [75]. In Pakistan, the overall income generation for utilization by the government and general public's day-to-day operations depends heavily on foreign remittances, which has a significant impact. Contribution in the form of remittances to the economy of Pakistan has provided clear evidence that overseas Pakistanis are playing a substantial role in the country's socioeconomic development as per the Pakistan Economic Survey, 2016–2017 [76]. [77] by applying the autoregressive-distributed lag (ARDL) model, have exposed the impact of the global output gap on workers' remittance inflows. They showed that exchange rate change had an insignificant effect from 1982 to 2016 in Pakistan. In another study [38], expressed concern over the stabilizing role of remittances by comparing it with other financial flows in Pakistan from 1974 to 2011 by utilizing the annual time series data. They revealed that flows of remittances increased as a relatively attractive source of external finance due to less volatility by surpassing the FDI for economic development and to manage the exchange rate shocks. Additionally, they claimed that remittance flows were remarkably resilient compared to other resource flows. Cismaș et al. (2020) reported a significant positive role of remittances in stabilizing the currency. A two-directional causal nexus among remittance, economic growth, and exchange rate, was observed by Ref. [78] for E7 economies. Fluctuations in economic development and remittance inflows can affect exchange rates. Exchange rates were used as a moderator in the nexus between remittance and economic growth by Ref. [79] as exchange rates play a crucial role in determining how remittances impact economic growth. Real exchange rate depreciations have a more significant positive effect on poverty through remittances [80]. This suggests that remittance inflows become more valuable in real terms when the local currency depreciates, potentially lifting more people out of poverty through increased income or consumption.

H4 Remittances have an effect on the exchange rate.

#### 2.3.2 Inflation and exchange rate

The impact of inflation on exchange rates depends on investment, trade dynamics, inflation expectations, interest rate differentials, and central bank policies. While high inflation can lead to currency exchange rate depreciation by eroding purchasing power and reducing international competitiveness, the relationship between inflation and exchange rates is complex and influenced by various economic factors. It was indicated by Ref. [81] that significant changes in inflation do have a nexus with exchange rate shocks [82]. specified the potential impact of inflation nexus with the exchange rate on money demand in Pakistan. A significant negative exchange rate estimate shows that the substitution effect overcomes the wealth effect during exchange rate variations [82].

In monetary finance theory, the influence of inflation on exchange rates is of utmost importance as it affects the cost of consumer products and has a ripple effect on other economic factors. Fluctuations in inflation rates have a vital role in determining monetary policy, impacting trade dynamics, and upholding economic stability, as suggested by the [7]. Inflation mostly influences currency rates by linking interest rates [83]. Changes in inflation rates, aim to either restrain or encourage consumer spending and investment, depending on the prevailing economic conditions. The objective of this policy response is to control inflationary pressures efficiently.

Countries that implement stricter monetary policies by raising inflation rates can attract foreign investments from investors looking for larger returns [10]. The surge of inflation results in an increase in demand for the native currency, which could potentially cause its value to rise. Countries that have greater inflation rates may see a decrease in the value of their currency. This happens because international investors sell off assets denominated in that currency to protect themselves from the loss of purchasing power [84]. The relationship between inflation and currency exchange rates is fundamentally intricate and fluid, entwining several economic factors. Clearly, comprehending this connection is essential for politicians, entrepreneurs, and investors as they traverse the decision-making process in a worldwide economic setting. This understanding allows them to use valuable information for strategic planning and managing potential risks.

H5 Inflation has an adverse effect on the exchange rate.

### 2.4 Conceptual Framework (determinants of currency depreciation)

**Fig. 2:**
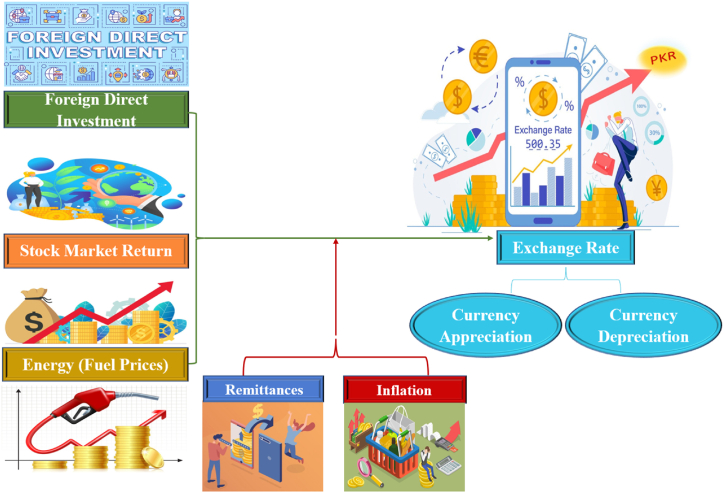
Conceptual framework of financial and energy determinants of exchange rate.

Fig. 2 presents a theoretical framework that illustrates the financial and energy variables that impact the exchange rate in Pakistan's economy. This framework demonstrates the influence of Foreign Direct Investment (FDI), as well as investor confidence as indicated by Stock Market Returns and fundamentals like Energy (Fuel Prices), on the exchange rate. The control variable remittances generally contribute to the appreciation of a currency exchange rate. Nevertheless, another control variable inflation can offset this impact, potentially resulting in the devaluation of the currency. Fig. 2 succinctly represents the economic storyline: when there is an influx of cash, which includes sustainable investments, and the stock market performs well, it typically indicates investor trust and can result in a more robust currency. On the other hand, expensive energy with high prices can put a lot of pressure on the exchange rate and decrease the currency's value. Remittances can serve as a stabilizing factor for the exchange rate. However, if the inflation rate is excessive, it might diminish purchasing power and result in currency depreciation of exchange rates. This paradigm facilitates comprehension of the intricate equilibrium that policymakers must uphold to promote investments that enhance economic growth and currency value while simultaneously regulating inflation and safeguarding against potential destabilization of the financial system caused by the influx of remittances. Fig. 2 demonstrates the conceptual link between the variables.

(Source: Developed by the author based on the literature review)

## 3 Research methodology

This study aims to determine the impact of independent variables foreign direct investment, stock market return, and energy fuel price on the dependent variables exchange rate change. Moreover, remittances and inflation are used as the control factors affecting decoding dynamics. This study used secondary monthly time-series data of all selected variables from July 2006 to June 2021.

The dependent variable is the change in the exchange rate as currency depreciation or appreciation was measured by taking the first difference in the exchange rate series and the change in USD$ rate for Pakistan currency. We have chosen to use the bilateral exchange rate as our dependent variable because it specifically represents the exchange rate between two currencies, which is relevant to our research focus on the relationship between the U.S. dollar and the Pakistani rupee. Proxy followed from Refs. [11,28,78]. The monthly USD$ rate for Pakistan currency was obtained from the official website of the State Bank of Pakistan (SBP) and then the current month minus the previous month divided by the current average monthly exchange rate was taken to measure the change in exchange rate.

Meanwhile, the stock index was collected from the website of the Pakistan Stock Exchange Limited (PSX) [42,52]. Stock market return (SR) was calculated as follows as shown in equation (1):

(1) $$R_{m,t}=\left(\frac{P_{m,t,}}{P_{m,t,-1}}-1\right)\times 100$$

Where R_m,t_ is the return at time t, P_m,t,_ and P_m,t−1_ are the market index prices at times t and t-1, respectively.

The monthly data of FDI [37,39], remittances [74,85], energy fuel prices [5,66] and inflation [54,66] were retrieved from the SBP website. All variables' data were collected from July 2006 to June 2021, considering all variable's similar selected panel observations. The measurement of variables is presented in Table 1 respectively.

**Table 1:** Measurement of variables.

Variables	Sign	Indicator/Source explanation
Exchange Rate		Change in the exchange rate (USD$ rate for Pakistan currency) as a proxy as indirect quotation bilateral exchange rate. Formulated as the current monthly average rate minus the previous monthly average rate divided by the current average monthly exchange rate [2,28,56,78]. Monthly average data gathered from the website of the State Bank of Pakistan www.sbp.org.pk/
Foreign Direct Investment	+/−	Monthly FDI inflows (USD$) in Pakistan, Data gathered from SBP [37,39].
Stock Market Return	−/+	The monthly average Stock market return is calculated as follows. $R_{m,t}=\left(\frac{P_{m,t,}}{P_{m,t,-1}}-1\right)\times 100$ (Previous month's average closing price of KSE100 index divided by current month's average closing price) minus 1 [52,56]. Data gathered from the website of the Stock Exchange of Pakistan psx.com.pk
Energy (Fuel Price)	−/+	Monthly Fuel crude prices per barrel in USD$. Data gathered from SBP [5,66,86].
Remittances	+	Monthly collection number of foreign remittances USD$ from overseas Pakistani. It was calculated as the log of remittances. Data gathered from SBP [74,85]
Inflation	−/+	Monthly Consumer Price Index and Data gathered from SBP [54,66].

For data analysis, both inferential and descriptive statistics were employed. First, data seasonality was checked using the Multicollinearity Test of Variance Inflation Factor and detailed diagnostics tests were applied, such as Breusch-Godfrey Serial Correlation L.M. test, LM test for Auto-regressive conditional heteroskedasticity, the heteroskedasticity test: Breusch-Pagan-Godfrey, the Breusch-Pagan/Cook-Weisberg test for heteroskedasticity, the Cameron & Trivedi's decomposition of IM-Test (White's test for (H0: homoskedasticity against Ha: heteroskedasticity, the Ramsey RESET test, Durbin-Watson Test Statistics, and Augmented Dickey-Fuller (ADF) root unit test. The tested variables had a different order of integration. Moreover, cumulative sums of recursive residuals (CUSUM) and cumulative sums of squares of recursive residuals (CUSUMSQ) were employed to check the stability and reliability [63,86].

Hence, the ARDL Bound Test, short-run and long-run tests, was used to test the effect of the independent variables on the dependent variable. The ARDL approach is a model for cointegration suggested by Ref. [87]. [63,86,88] have expressed that the ARDL approach to cointegration is preferable to other conventional cointegration methods due to some advantages of this model, such as it can take a wide range of lag numbers captured in the data-generating process. Notably, in a more general-to-specific approach of ARDL modeling framework, ARDL can be applied in all cases whether the series are mutually cointegrated or I (0), I (1). In addition, the ARDL technique uses lag for establishing long-term relationships, which makes this analysis technique more suitable.

In this study, overall Stata-17, EViews version 9, and OriginPro-2023 statistical software were utilized. ARDL EViews software was chosen due to its feature of automatically numbering the lag selection of variables to correct for serial correlation [86,89]. The association was used to verify the relationship between the variables [90]. used the ARDL model and showed that this model presented effective and reliable results in Egypt. In Ghana [54], also conducted a study to determine the relationship between stock return and exchange rate using the ARDL estimation technique.

In Bangladesh [26], and in Pakistan [86] used the analytical cointegration technique and ADF tests to examine the association between exchange rate and economic growth using variables like foreign investment, and different government indicators, which represented monetary policies such as expenditures and consumption. Exchange rate management is a significant policy objective to achieve diverse goals and maintain external competitiveness. Ellahi (2011) used the ARDL model in Pakistan to examine the relationship between exchange rate instability and FDI behavior from 1980 to 2010. Besides that [77], used the ARDL approach to cointegration for an empirical assessment of exchange rate movement and workers' remittance inflows. Hence, based on the literature review and discussion, the econometric model by adopting [86,91] is as follows shown in equation (2):

Equation (2) of the econometric long-run model is as follows:

(2) $${ER}_{t}=\eta_{0}+\eta_{1}{ER}_{t-1}+\eta_{2}{FDI}_{t-1}+\eta_{3}{SMR}_{t-1}+\eta_{4}{Energy}_{t-1}+\eta_{5}{REM}_{t-1}+\eta_{6}\;{\mathrm{INF}}_{t-1}+\sum_{j=1}^{p}\phi_{1j}\Delta {ER}_{t-j}+\sum_{j=0}^{q}\phi_{2j}\Delta {FDI}_{t-j}+\sum_{j=0}^{q}\phi_{3j}\Delta {SMR}_{t-j}+\sum_{j=0}^{q}\phi_{4j}\Delta {Energy}_{t-j}+\sum_{j=0}^{q}\phi_{5j}\Delta {rem}_{t-j}+\sum_{j=0}^{q}\phi_{6j}\Delta \inf_{t-j}+\xi_{t}$$

To determine whether there is a long-term link between the variables, this study initially utilized the Wald test, especially employing an F-test to assess the combined significance of the coefficients of the lagged variables. We conducted a test on the null hypothesis H0: η1 = η2 = η3 = η4 = η5 = η6 = 0, which suggests that there is no cointegration. We compared it to the alternative hypothesis Ha ≠ η1≠ η2 ≠ η3≠ η4≠ η5≠ η6≠ 0. Subsequently, it is necessary to compute the immediate consequences based on the error correction model which is shown in equation (3).

(3) $$\Delta {ER}_{t}=\sum_{j=1}^{p}\lambda_{1j}\Delta {ER}_{t-j}+\sum_{j=0}^{q}\lambda_{2j}\Delta {FDI}_{t-j}+\sum_{j=0}^{q}\lambda_{3j}\Delta {SMR}_{t-j}+\sum_{j=0}^{q}\lambda_{4j}\Delta {Energy}_{t-j}+\sum_{j=0}^{q}\lambda_{5j}\Delta {REM}_{t-j}+\sum_{j=0}^{q}\lambda_{6j}\Delta {\mathrm{INF}}_{t-j}+{k\xi }_{t-1}+e_{t}$$

Where, β0 = constant, FDI = foreign direct investment, SMR = stock market return, Energy as energy fuel prices, Rem = remittances, INF = inflation, and μ = error term. λ is used to estimate the short-run effects if the value of k is significant and statistically negative.

The study models take into cognizance the effect these variables can have on the exchange rate in Pakistan using a re-estimated ARDL with an alternate variables model. Therefore, a robust model is estimated without inflation variables to confirm the validity of the key variables.

## 4 Results and discussion

### 4.1 Evaluation of graphical presentation

**Fig. 3:**
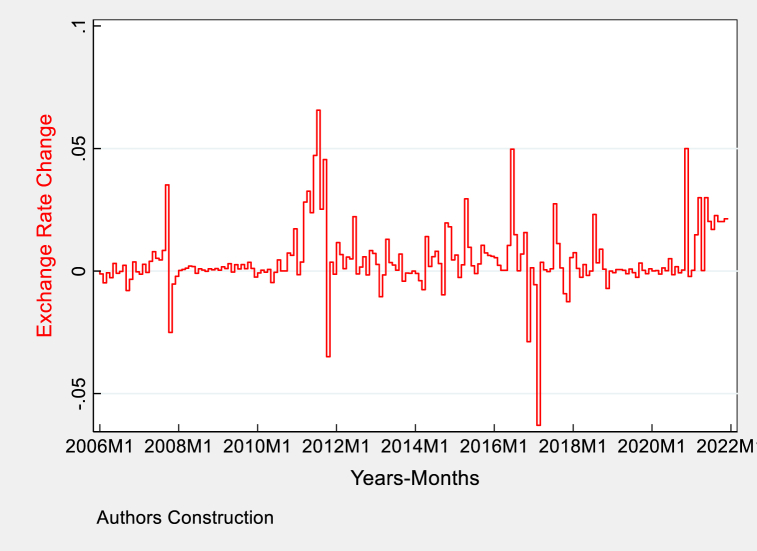
Graphical trends of exchange rate change in Pakistan.

**Fig. 4:**
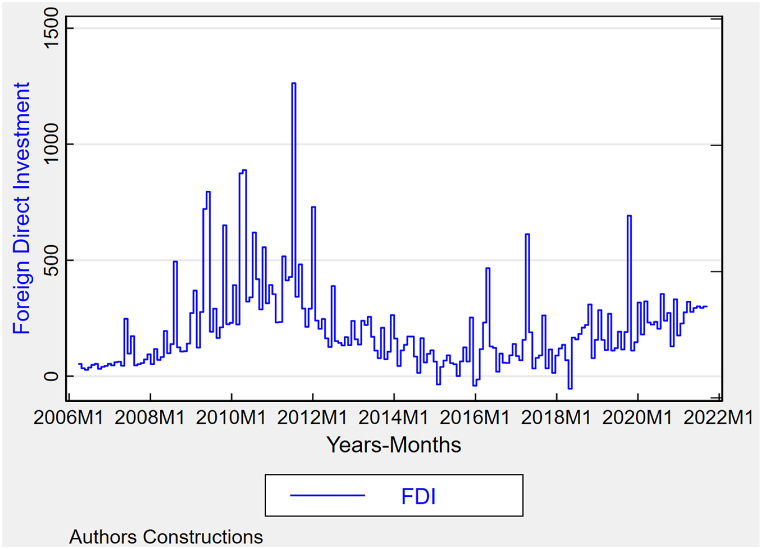
Graphical trends of FDI in Pakistan.

**Fig. 5:**
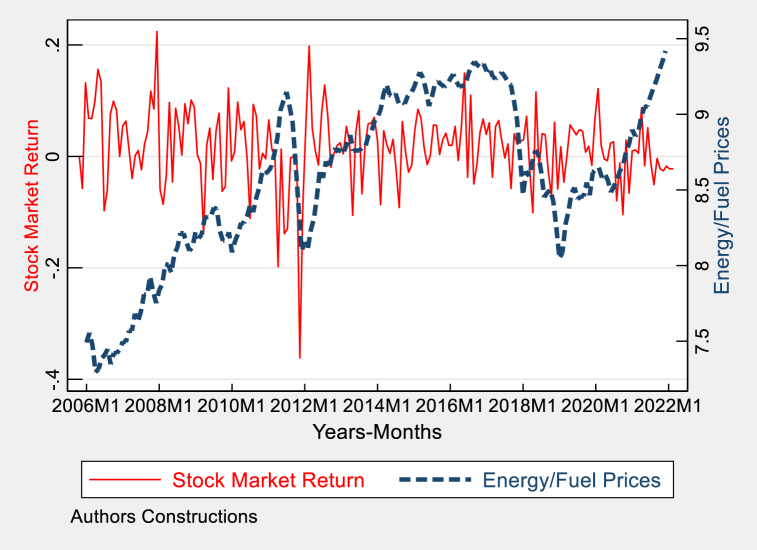
Graphical trends of stock market returns and energy fuel prices in Pakistan.

**Fig. 6:**
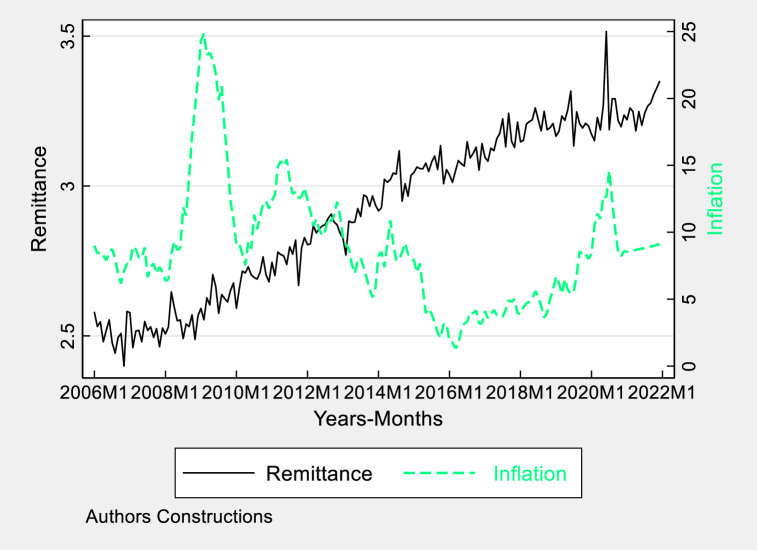
Graphical trends of remittances and inflation in Pakistan.

**Fig. 7:**
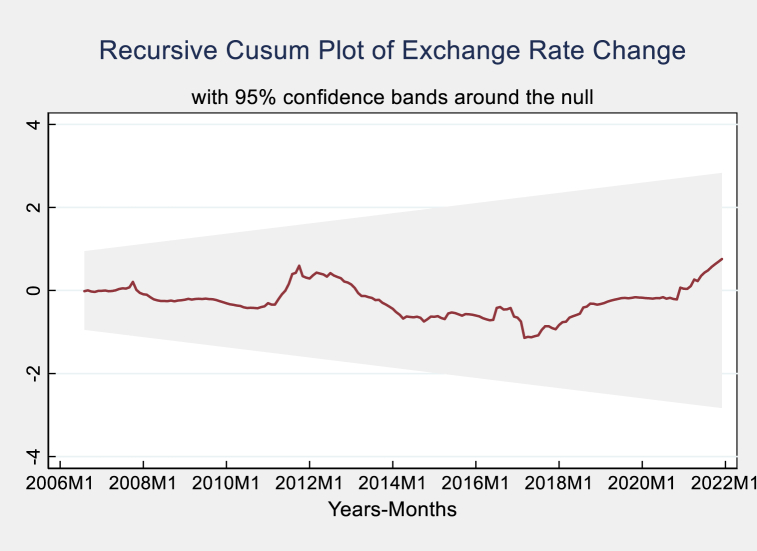
The plot of recursive CUSUM for exchange rate change.

The graphical trends of study variables are demonstrated below in Fig. 3, Fig. 4, Fig. 5, Fig. 6, Fig. 7 for the monthly period of 2006–2021. The graph in Fig. 3 illustrates the variations in Pakistan's exchange rate, showcasing currency exchange rate devaluation and appreciation patterns. Significantly, the exchange rate trends undergo multiple fluctuations over time, which may align with times of economic volatility or expansion, policy modifications, or foreign economic disturbances. Based on the visual data presented in Fig. 3, it is evident that there are moments of significant decline, particularly noticeable during specific time intervals where the extent of the change is more prominent. The depreciation occurrences may be associated with macroeconomic difficulties, such as political concerns, fluctuations in global commodity prices, or fiscal and monetary policy modifications.

In contrast, periods of appreciation, although less dramatic, suggest that the economy may have been strengthened by favorable factors such as higher levels of FDI, and a significant increase in remittances helping improve trade balances. These patterns are important for economic study because they demonstrate how the Pakistani rupee as of USD $ exchange rate is affected by both internal economic activities and international economic developments. The Pakistan currency USD$ exchange rate has experienced significant fluctuations, highlighting the need for robust economic policies to protect against external disruptions and encourage sustainable economic activities. Policymakers must comprehend these trends to formulate strategies that stabilize the exchange rate, creating a conducive climate for investment and economic growth The Fig. 3 show exchange rate change over the period.

The investment trends demonstrated in Fig. 4, particularly Foreign Direct Investment (FDI) in Pakistan, can potentially impact the currency rate. The surges in FDI are expected to result in an increase in the value of the domestic currency as a result of the inflow of foreign capital. The more consistent but smaller increases in FDI indicate a growing interest in sustainable development. While this may not result in immediate advantages in exchange rates, it can contribute to long-term economic stability and may lead to a stronger currency in the future. The association between these investment flows, and the exchange rate highlights the necessity of economic policies that utilize the advantages of FDI for immediate economic expansion while fostering FDI to support long-term appreciation of the currency in line with environmental objectives. The Fig. 4 show graphical trend of FDI in Pakistan.

Fig. 5 presents a side-by-side comparison of the fluctuations in Pakistan's stock market returns and energy/fuel prices. The stock market returns display significant volatility, which is influenced by investor sentiment and economic situations. Periods characterized by high returns have the potential to attract foreign investment in energy, which in turn can lead to an increase in the currency's value. Also, elevated energy prices can have two main consequences: they can raise production expenses and influence the balance of payments, frequently resulting in currency devaluation. Alternatively, they may also indicate strong market returns, which can attract investments and bolster the currency's value. These trends are directly relevant to the exchange rate because they impact on the movement of foreign investment, energy prices, and the country's stock market returns, which are important factors in determining the currency's strength.

Fig. 6 depicts the patterns of remittances and inflation in Pakistan, showing a consistent increase in remittances. This growth indicates a growing amount of money being sent back to Pakistan by expatriates, which supports the local currency by increasing the availability of foreign exchange however this can lead to dependence of the economy on external factors to some extent if weak foreign policy becomes weak. Nevertheless, the line with dashes representing inflation has peaks that frequently align with remittance surges, implying that substantial influxes of currency can also contribute to inflation, perhaps resulting in gradual currency devaluation. The influence of remittances on the exchange rate is twofold, as they can stabilize and destabilize the local currency. This outcome is determined by various economic factors, including the country's monetary policy and its ability to handle these inflows without causing inflation.

### *4.2* Baseline analysis results

Descriptive statistics have described all the variables in this study. For example, the mean values have explained the measure of central tendency. The Std. Dev. test indicated that the data had a normal distribution given variation or dispersion in a set of values. Table 2 shows the detailed descriptive statistics results.

**Table 2:** Descriptive statistics results.

Variable	Obs	Mean	Std. Dev.	Min	Max
ER	192	0.004	0.013	−0.063	0.066
FDI	192	5.001	0.901	−0.452	7.141
SR	192	0.017	0.068	−0.362	0.224
Energy	192	8.579	0.548	7.295	9.418
REM	192	2.916	0.271	2.399	3.515
INF	192	8.806	4.533	1.324	24.858

Table 3 presents the correlation between this study's dependent and independent variables. The association between FDI and the exchange rate was 17.4 %, at a significant level of 1 %, suggesting that FDI positively correlated with the exchange rate. This result is in line with (Pantelopoulos, 2023). Thus, the relationship between stock market returns and currency depreciation was negative (23.9 %). This finding concurs with the findings of [55] in Pakistan, who suggested that this relationship is related to the contraction of a country's economy. This indicates that Pakistan uses currency deposits to pay external debts such as the International Monetary Fund (IMF) and commercial and sovereign debts. Furthermore, indicators have shown instability in the financial markets and lower sustainable socio-economic development in Pakistan. Next, the hypothesis that Energy as fuel prices, remittances, and inflation significantly correlate with the exchange rate was accepted and endorsed. There was a 23.4 %, 8.4 %, and 7.3 %, correlation between energy fuel prices, remittances, and inflation, respectively, with exchange rate. Overall, all the variables' values are less than 80 %, as followed by [91, 86} which means there are no collinearity issues as outcomes are shown in Table 3.

**Table 3:** Pairwise correlations results.

	Variables	(1)	(2)	(3)	(4)	(5)	(6)
(1)	ER	1.000					
(2)	FDI	0.174∗	1.000				
		(0.016)					
(3)	SR	−0.239∗	−0.132∗	1.000			
		(0.001)	(0.067)				
(4)	Energy	0.234∗	0.098∗	−0.125∗	1.000		
		(0.001)	(0.175)	(0.085)			
(5)	REM	0.084∗	0.043	−0.124∗	0.708∗	1.000	
		(0.244)	(0.558)	(0.086)	(0.000)		
(6)	INF	0.073∗	0.413∗	−0.121∗	−0.285∗	−0.441∗	1.000
		(0.316)	(0.000)	(0.093)	(0.000)	(0.000)	

Table 4 shows the results of a Variance Inflation Factor (VIF) test to ensure the degree of multicollinearity among various variables used in a regression model. The VIF test is crucial for verifying that the regression coefficients obtained are reliable, as high multicollinearity among variables can distort these estimates. According to the results reported in Table 4 indicate that values are well below the common threshold of 5 [63] which indicates problematic multicollinearity, suggesting no multicollinearity. These findings imply that the model is robust in terms of multicollinearity, ensuring the stability and accuracy of the regression coefficients. This enables clear, unbiased interpretations of how each independent variable influences the dependent variable, providing valuable insights for analysis.

**Table 4:** Results for multicollinearity test: Variance inflation factor.

Variables	VIF	1/VIF
FDI	2.390	0.419
SR	1.570	0.636
Energy	1.260	0.792
REM	1.070	0.938
INF	1.030	0.972
**VIF**	**1.464**	1/VIF

Table 5 tabulates the unit root test results, whereby the A.D.F. test was used in line with [92] one-sided p-values. A.D.F. test findings indicated that the data for all the variables stationary i.e., Exchange Rate, Stock Market Return and Inflation were stationary at the level while FDI, Energy (Fuel Price) and Remittance were stationary at first difference.

**Table 5:** Unit root test results.

Variables	T-Statistic	Prob.∗	1 % level	5 % level	10 % level
**At Level (Lag Length: 0)**					
Exchange Rate	−10.50	0.000	−3.47	−2.88	−2.58
FDI	−2.29	0.18	−3.47	−2.88	−2.58
Stock Market Return	−11.80	0.000	−3.47	−2.88	−2.58
Energy (Fuel Price)	−1.97	0.30	−3.47	−2.88	−2.58
Remittance	−0.80	0.82	−3.47	−2.88	−2.58
Inflation	−0.51	0.01	−3.47	−2.88	−2.58
**At First Difference**					
FDI	−11.78	0.00	−3.47	−2.88	−2.58
Energy (Fuel Price)	−8.64	0.00	−3.47	−2.88	−2.58
Remittance	−17.55	0.00	−3.47	−2.88	−2.58

### 4.3 Co-integration test of ARDL Bound Test

The study in Pakistan employed the ARDL bounds testing approach by following [93,94] to investigate the enduring connections among different economic factors. Our analysis validates cointegration among the variables studied. Table 6 shows an F-statistic value of 14.564, which exceeds the upper critical barrier of 2.95 at the 10 % significance level. We reject the null hypothesis of no cointegration and support the alternative hypothesis, showing considerable interdependencies across vital economic indices [86,91]. as suggested by these studies.

**Table 6:** Results of the ARDL bound test.

Estimation model	k	F-Statistics (Value)	Level of significance	Critical bound F-statistics Lower Upper	
			Asymptotic: n = 1000		
F-statistic	6	14.564	10 %	1.98	2.95
			5 %	2.27	3.28
			2.5 %	2.55	3.61
			1 %	2.88	3.99
Actual Sample Size (192)			Finite Sample: n = 80		
			10 %	2.088	3.103
			5 %	2.431	3.518
			1 %	3.173	4.485

Null Hypothesis: No levels of relationship.

### 4.4 ARDL approach results

This study applied the ARDL model to examine the determinants of exchange rates in Pakistan from July 2006 to June 2021. The ARDL model results in Table 7 provide valuable comparisons between the short-term dynamics and long-term linkages among crucial financial and energy indicators that affect the exchange rate and Pakistan's economic stability. Table 5 lists the ARDL model short-run and long-run results, whereby the values of R-squared and adjusted R-squared are 0.452 and 0.443, respectively, which indicate the goodness of fit. Next, the F-statistic was 20.341, which showed that the estimated model was significant, while the Durbin-Watson statistic result was 2.875, implying no autocorrelation in the data. The best model (1, 0, 0, 1, 0, 1, 0) was selected based on the Akaike information criterion (A.I.C.).

**Table 7:** Result of ARDL short and long run model.

				
Variable	Coefficient	Std. Error	t-Statistic	Prob.
Short-Run Result by Using Error Correction Model				

Constant	0.026	0.002	13.000	0.000
Ex _(1)_	−0.903	0.069	−13.087	0.000
FDI	0.092	0.130	0.708	0.131
FDI (−1)	0.035	0.001	35.000	0.002
SR	−0.033	0.013	−2.538	0.014
LNFUEL	0.009	0.004	2.250	0.011
REM	−0.007	0.084	−0.083	0.187
REM (−1)	−0.036	0.014	−2.571	0.014
INF	−0.002	0.003	−0.667	0.269
INF (−1)	−0.006	0.008	−0.750	0.218

Long-Run				

C	−0.025	0.09	−0.278	0.005
FDI	0.034	0.009	3.778	0.002
SR	−0.039	0.016	−2.438	0.016
LNFUEL	0.017	0.007	2.429	0.011
REM	−0.012	0.007	−1.714	0.061
INF	−0.016	0.023	−0.696	0.213

R-squared	0.452			
Adjusted R-squared	0.443			
F-statistic	20.341			
Probe(F-statistic)	0.000			
Durbin-Watson stat	2.875			

Selected Model: ARDL (1, 0, 0, 1, 0, 1, 0)				

According to the Error Correction Model (ECM) in Table 7, there is a negative link between the exchange rate and its coefficient of −0.903 in the short run. However, the t-statistic of −13.087 is statistically significant with a p-value of less than 1 %. This implies that short-term changes in the exchange rate may exhibit a major degree of responsiveness to deviations from its previous values during a specific period. Exchange rate change results showed that the currency deprecation had a lag effect, which was significant but negative in the short run. Our findings endorse the concluding conclusions of [34] those who have reported a declining inclination of Asian countries' currency exchange rates in the long run.

In the short run, the preceding periods without lag FDI show a positive but insignificant effect on the exchange rate. Thus, with lag length 1 FDI shows a significantly positive effect on the exchange rate which indicates the in-short run FDI affects a 3.5 % change in exchange rate with a significance level of 1 %. Moreover, in the long-run findings demonstrate more enduring associations. FDI continues to exert a significant positive influence, although with a slightly reduced 3.4 %, indicating a consistent and favorable effect on exchange rate stability as economic growth through currency appreciation over a period of time. This suggests that FDI inflows have a positive effect on the short-term fluctuations in FDI levels and improve the stability of the exchange rate. This suggests that immediate changes in FDI levels may reliably anticipate economic behavior. The FDI and exchange rate stability results align with [39], who recommended that FDI and international trade positively affect exchange rate links in developing countries.

The stock market return had a negative and significant role in the exchange rate as currency depreciation in both the short- and long-term 3.3 % and 3.9 %, respectively with a significance level (0.01). Thus, aligned with [55] as they also reported similar findings. The variable energy prices (log value of fuel) demonstrate a significantly positive connection in the immediate period indicating 0.9 % in the short run while 1.7 % in the long run at the significance level (0.011), suggesting that energy usage is crucial in stimulating economic activity, even in the near term. The current findings are aligned and endorse the conclusion of [65] which has shown that in small open economies, fuel prices are expected to significantly impact the currency's relative value because changes in oil prices have substantial implications on the demand and supply of foreign exchange. The findings are aligned with [43]. These findings align with their short-term effects and underscore their crucial roles in shaping the structural behavior of the economy.

The control variable remittances (REM) exhibit a negative but insignificant effect on the exchange rate. But with lag length 1 in short-run and long-run remittances show a negative but significant effect on the exchange rate which indicates remittances affect (0.036 % and 0.012 %) change exchange rate with a significance level of 1 % and 5 % respectively in the in-short run and long run. For remittances, it had a strong negative and considerable role in currency depreciation, but in the long run, it became a weak negative role. This suggests that an increase in remittances could potentially have a temporary dampening impact on the relevant variables. This could be attributed to adjustments in exchange rates or inflationary pressures. Furthermore, inflation has a negative effect, although it is not large and insignificant. Remittances (REM) and inflation (INF) both indicate the intricate relationship between external monetary inflows and price stability and their influence on the exchange rate economy over protracted periods. This implies that a change in inflation will lead to a rise in the exchange rate in the short and long term and these findings are linked with [36].

The transition from being unimportant in the short term to being important in the long term could suggest FDI, Stock returns, energy prices and hidden exchange rate economic patterns that are only apparent over lengthy timeframes. This result may be due to Pakistan utilizing foreign currency to meet the external, commercial, and sovereign debt payments in the short run and to finance imports using browning. In recent research [95], showed that investment spending and exchange rate depreciation have a contractionary impact. Nonetheless, a reduction in the exchange rate can have a relationship with the long-run deficit issued in the domestic currency to boost investments. In summary, these findings emphasize the significance of considering both immediate variations and extended patterns when developing economic strategies in Pakistan. These policies should strive to leverage the beneficial effects of foreign direct investment (FDI) and effectively handle the intricacies of government expenditure, energy usage, and remittance inflows to cultivate a stable and expanding economy given exchange rate fluctuations. The Results of the ARDL model are shown in Table 7 for the short run by using Error Correction Model and the long run model.

### 4.5 Results of diagnostics and robustness check analysis

#### 4.5.1 CUSUM and squares of the cumulative sum of recursive residuals

**Fig. 8:**
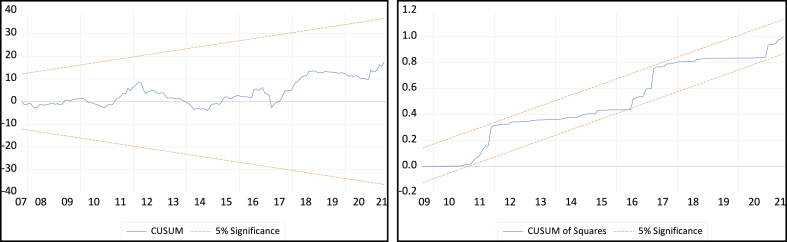
The plot of CUSUM and Squares of the Cumulative Sum of Recursive Residuals in the study.

This study utilized the technique introduced by Ref. [96] which employs the cumulative sum of recursive residuals (CUSUM) and the cumulative sum of squares of recursive residuals (CUSUMSQ) to evaluate the stability of statistical models. Fig. 6, Fig. 7 demonstrate the model's resilience in response to fluctuations in the exchange rate and study. Both Figures illustrate that the recursive CUSUM and CUSUMSQ plots constantly stay within the upper and lower critical bounds, suggesting that the model retains its stability at a significance level of 5 %. The persistent placement of the CUSUM and CUSUMSQ plots inside these crucial boundaries confirms the model's stability and durability, indicating that the model consistently adjusts to both short-term variations and long-term trends in exchange rate data and research models. The model's ability to adhere to the limits in different situations demonstrates its effectiveness and efficiency in accurately representing the fundamental movements of exchange rates. The capacity of the CUSUM and CUSUMSQ methodologies to verify the stability and persistence of the model is essential for economic and financial analysis [86,91]. The model's trustworthiness for predicting and policy-making is guaranteed by its capacity to reliably recognize systematic changes and respond to random fluctuations within the stated limits, as shown in Fig. 7, Fig. 8.

### 4.6 Results of Diagnostics Tests and robustness check method

Different diagnostics tests were performed and demonstrated in Table Appendix-A and Appendix-B to avoid autocorrelation and multicollinearity. Firstly, the Lagrange Multiplier (L.M.) test was used to analyze the correlation between the independent and dependent variables. The most helpful test for serial correlation would be the Breusch-Godfrey LM test and the LM test for Auto-regressive conditional heteroskedasticity (ARCH). Based on the L.M. tests values showed no evidence of autocorrelation in residuals. Furthermore, Breusch-Pagan-Godfrey and Breusch-Pagan/Cook-Weisberg test results revealed no heteroskedasticity in the model because the probability ensures the standard. Moreover, IM-test, Ramsey RESET, and Durbin-Watson Test Statistics endorse the model's fitness to use. The diagnostics test results are shown in Table Appendix-A.

We conducted a robustness check of our short and long-run analysis using the alternate variables by re-estimating the approach, as shown in Table Appendix-A. These results support the conclusions obtained using the ARDL technique of key variables, which aligns with previous research by Refs. [86,91]. Additionally, the short and long-run elasticities measured in Table 7 are consistent with our findings, increasing our results' reliability and predictability for policymakers. This robustness check not only confirms the durability of our long-term linkages but also emphasizes their significance and practicality in guiding successful policy interventions targeted at promoting economic stability and prosperity in Pakistan. It highlights that as FDI, stock market return and energy-using fuel prices expand, it can contribute to a more stable and perhaps increasing exchange rate as stated in the hypothesis. This arises from the notion that a country attracts more foreign investment, energy use and market return and enhances exchange rate stability and economic growth, hence fortifying the national currency. Using ARDL, which addresses data issues such as non-stationarity and endogeneity, this study establishes accurate relationships. It provides dependable evidence that depending on the directional movements of the variables, the effect on both the environment and the country's economic stability, including its exchange rate dynamics can be predicted. This study's findings are crucial for policymakers who want to improve economic sustainability and attract foreign investment by encouraging environmentally conscious development [86].

### 4.7 Discussion of findings

The ARDL model provides a comprehensive examination of the intricate relationships between financial factors, including foreign direct investment (FDI), stock market returns (SR), and energy prices (LNFUEL) on the exchange rate in Pakistan's economy. The independent factors affecting the exchange rate, which is the dependent variable consider the remittances (REM), and inflation (INF) as control variables. The findings suggest complex linkages in which each element has a distinct impact on the currency's exchange rate movement, both in the short and long term. The association between FDI and energy prices with the exchange rate is significantly positive, indicating that they have a role in strengthening the Pakistan exchange rate by increasing the demand for exchange rates and raising renewable energy production because of the rise in fuel costs. This aligns with economic theories that propose Foreign Direct Investment (FDI) as a catalyst for financial growth, enhancing the balance of payments, and consequently bolstering the domestic currency. The impact of foreign direct investment (FDI) on currency exchange rates is influenced by various factors such as the magnitude of the investment, the sectors in which it is made, the financial situations of the nations concerned, local regulations regarding FDI, investor attitudes, and worldwide market trends [97]. FDI inflows typically increase the value of the currency rate, indicating a positive perception of the country's financial prospects and fostering stability. On the other hand, substantial outflows might lead to a decrease in value due to changes in investor trust or financial disturbances. FDI movements inherently impact currency valuations, as acknowledged in financial research. This emphasizes the significant significance of foreign direct investment (FDI) in determining exchange rate dynamics. FDI in Pakistan has a favorable long-term relationship with the exchange rate, affecting external debt dynamics [97,98].

Energy exhibits a positive effect in both short and long term, possibly due to energy usage is crucial in stimulating economic activity, even in the near term. The current findings are aligned and endorse the conclusion of [65] which has shown that in small open economies, fuel prices are expected to significantly impact the currency's relative value because changes in oil prices have substantial implications on the demand and supply of foreign exchange. The findings are aligned with [43]. These findings align with their short-term effects and underscore their crucial roles in shaping the structural behavior of the economy. Green energy as promoting renewable energy an understanding of the early adverse effects of green energy investments, as foreign investors may reallocate their FDI portfolios away from these assets if they perceive more risks or lower immediate returns, resulting in currency devaluation [44].

Moreover, stronger stock market returns and more remittances contrary to expectations do not necessarily support currency appreciation [62]. These factors may alternatively imply speculative investments or contribute to inflationary pressures that could cause the currency to depreciate. The inverse correlation between currency value and stock market returns in the near term suggests that an increase in stock market profits does not automatically attract foreign investment that promotes exchange rate currency appreciation. Alternatively, it could indicate a speculative investment climate or the repatriation of profits by foreign investors, resulting in the outflow of money. The relationship between stock market return and exchange rate fluctuations is crucial. The model's results demonstrate that both stock market returns (SR) and changes in energy prices influence the exchange rate, highlighting the significance of FDI. The findings are aligned with [20]. Findings suggest that significant gains in the stock market can potentially attract foreign investment, strengthening the currency. However, this effect may be reversed if accompanied by substantial profit repatriation or speculative investment. In the same way, elevated energy prices can impact the trade balance and, consequently, the exchange rate.

Conversely, the results suggest that the exchange rate is negatively affected by inflation may be due to the reduced international purchasing power of the domestic currency caused by excessive inflation [82]. Inflation provides help in predicting exchange rate changes [99]. Hence, this intricate situation highlights the crucial equilibrium required in policy formulation to successfully use foreign investments and effectively handle financial and energy factors such as inflation and energy expenses. The findings endorse attaining improving currency stability while preserving sustainable development, as vital for enduring economic growth and stability in Pakistan [34,35]. suggested that economic stimulus through quantitative easing policy resulted in a long-run decline in Asian countries' currencies and the need to focus on a strong and robust regularity system for the exchange rate fluctuations.

## 5 Conclusion and policy inferences

The research aims to examine the financial and energy determinants of the exchange rate in Pakistan from July 2006 to June 2021 with the monthly dataset. In this study, variable exchange rate change, such as currency depreciation or appreciation is observed as a dependent variable. The determinants as independent variables were foreign direct investment (FDI), stock market return (SR), and energy (fuel prices). In this study, the control variables are remittances and inflation. This study in the first step evaluates the graphical trends of key variables to understand the monthly fluctuations and data nature better. Secondly, this study checked the data's seasonality by employing the baseline and diagnostic tests, which showed that the data were stationary at different levels as well as model fitness, accuracy, and reliability. Next, in the final step, ARDL Bond test, CUMUM, ARDL short-run as well and long-run regression were used to test the effect of the independent variables on the dependent variable. The correlation between variables indicates that all the variables have positive effects, however, SR has a negative correlation with the exchange rate.

Meanwhile, the ARDL model results showed that the currency exchange rate had lag effects, which were significant but negative. For FDI, it had a significantly substantial and positive role in the exchange rate as currency depreciation in the short- and long-term at lag. Next, the stock market return had a negative and significant role in the exchange rate as currency depreciation in both the short- and long-term. Also, energy fuel prices have positively and significantly contributed to changes in exchange rates in Pakistan in the short and long run. In contrast, remittances had an active negative and considerable role and a weak negative role in the exchange rate as currency depreciation in the short and long run. Lastly, inflation had a decisive negative but insignificant role in the short and long term. Hence, the study hypothesizes that FDI is endorsed to strengthen the exchange rate. Conversely, stock market declines might exert a negative impact due to concerns over their short-term profitability, increased energy fuel prices, and high inflation are associated with high exchange rate changes as currency depreciation. Contrary to typical expectations, remittances also show a complex relationship with the exchange rate, suggesting potential adverse effects if not properly managed.

The findings emphasize the intricate interaction of these factors and the necessity for focused financial strategies that encourage consistent and sustainable investment streams, specifically in green renewable energy sources, to strengthen Pakistan's overall exchange rate financial stability and currency robustness. To conclude, this study balanced growth policy implications as fast changes in the exchange rate as currency depreciation due to a reduction in FDI, lower stock market returns, and an increase in energy fuel prices have become among the most critical issues in Pakistan. All the stakeholders need to make a concerted effort to combat the menace of financial and energy crises in Pakistan. The objective should be to attract FDI with a focus on green investment, explore more oil reserves, shift towards green and renewable energy based on advanced carbon capture technology within the country, and attract remittance. Moreover, to stabilize the exchange rate in Pakistan, the government needs to effectively manage the exchange rate and regulatory system to attract foreign and local investors. Compared to China, Malaysia, India, Bangladesh, and Indonesia, trade promotions in Pakistan are focused on the least. Thus, a stringent policy must be put in place to minimize changes in the exchange rate in Pakistan. More attention should be paid to proper and appropriate trade strategies, promotions, and policies. This study's results will be helpful for policymakers and academicians worldwide. Future studies could explore how political stability influences FDI and the subsequent moderating role of exchange rate and carbon capture technology on the long-term effects of financial volatility, especially in developing countries like Pakistan. Subsequent research should also focus on elucidating the factors that influence the relationship between energy prices and currency rates and the resulting impact on investment, trade, and economic stability. This will offer significant insights for policymakers, businesses, and investors.

## CRediT authorship contribution statement

**Saif Ullah:** Writing – original draft, Visualization, Validation, Methodology, Investigation, Formal analysis, Data curation, Conceptualization. **Haitham Nobanee:** Writing – review & editing, Visualization, Validation, Supervision, Methodology, Formal analysis, Conceptualization.

## Data availability statement

The data will be available upon request.

## Declaration of generative AI and AI-assisted technologies in the writing process

During the preparation of this work the author(s) used Grammarly Software (https://app.grammarly.com/) in order to avoid errors in the English language. After using this tool/service, the author(s) reviewed and edited the content as needed and take(s) full responsibility for the content of the publication.

## Funding sources

This research did not receive any specific grant from funding agencies in the public, commercial, or not-for-profit sectors.

## Declaration of competing interest

The authors declare the following financial interests/personal relationships which may be considered as potential competing interests:The authors of this manuscript, Saif Ullah and Haitham Nobanee, currently serve as Associate Editors for Heliyon. The authors declare no other known competing financial interests or personal relationships that could have appeared to influence the work reported in this paper. If there are other authors, they declare that they have no known competing financial interests or personal relationships that could have appeared to influence the work reported in this paper.

## Figures and Tables

**Table Appendix-A:** Results of Diagnostics Tests

a. Results of the Breusch-Godfrey Serial Correlation L.M. test			
F-statistic	0.186361	Prob. F (11,192)	0.8301
Obs∗R-squared	0.402533	Prob. Chi-Square (2)	0.8177

**b. LM test for Auto-regressive conditional heteroskedasticity (ARCH)**			

Lags (p)	CHI2	df	Prob. >Chi2
1	1.688	1	0.1939
H0: No ARCH effects vs.		H1: ARCH (p) disturbance	

**c. Results of the heteroskedasticity test: Breusch-Pagan-Godfrey**			

F-statistic	1.273683	Prob. F (11,192)	0.2433
Obs∗R-squared	13.85908	Prob. Chi-Square (11)	0.2409
Scaled explained SS	62.38135	Prob. Chi-Square (11)	0.0000

**d. Breusch-Pagan/Cook-Weisberg test for heteroskedasticity**			

F (6, 192) =	1.61	Prob > F =	0.1454

**e. Cameron & Trivedi's decomposition of IM-Test (White's test for (H0: homoskedasticity against Ha: heteroskedasticity**			

source	CHI2	df	p
Heteroskedasticity	23.37	27	0.6647

**f. Results of the Ramsey RESET test**			

	**Value**	**df**	**Probability**
t-statistic	4.720411	192	0.0000
F-statistic	22.28228	(1, 192)	0.0000
Likelihood ratio	22.53782	1	0.0000

**g. Durbin-Watson Test Statistics**			

Durbin-Watson d-statistics (7, 192)		1.701635	between 2 and 1 so ok

**Table Appendix-B:** Results of ARDL Robustness Check with Alternate Variables and After HAC

Variable	Coefficient	Std. Error	t-Statistic	Prob.
Short-Run Robustness with Alternate Variables After HAC				

Constant	0.027	0.003	9.000	0.000
Ex_(1)_	−0.814	0.055	−14.800	0.000
FDI	0.087	0.120	0.725	0.116
FDI (−1)	0.042	0.002	21.000	0.001
SR	−0.029	0.011	−2.636	0.014
LNFUEL	0.011	0.005	2.200	0.030
REM	−0.008	0.106	−0.075	0.186
REM (−1)	−0.034	0.014	−2.429	0.010

Long-Run with Alternate Variables After HAC				

C	−0.028	0.004	−7.000	0.004
FDI	0.032	0.011	2.909	0.001
SR	−0.041	0.015	−2.733	0.014
LNFUEL	0.016	0.008	2.000	0.013
REM	−0.009	0.005	−1.800	0.067

R-squared	0.431	S.E. of regression		0.0112
Adjusted R-squared	0.428	Sum squared resid		0.0196
F-statistic	19.876	Log likelihood		572.7575
Probe(F-statistic)	0.000	S.D. dependent var		0.0130
Durbin-Watson stat	1.987	Akaike info criterion		−6.0083

Selected Model: ARDL (1, 1, 0, 0, 0, 1)				
After HAC Case 2: Restricted Constant and No Trend				
Model selection method: Akaike info criterion (AIC)				
HAC standard errors & covariance (Bartlett kernel, Newey-West fixed bandwidth = 5.0000)				
